# Intelligent Emotion and Sensory Remote Prioritisation for Patients with Multiple Chronic Diseases

**DOI:** 10.3390/s23041854

**Published:** 2023-02-07

**Authors:** A. H. Alamoodi, O. S. Albahri, A. A. Zaidan, H. A. Alsattar, B. B. Zaidan, A. S. Albahri, Amelia Ritahani Ismail, Gang Kou, Laith Alzubaidi, Mohammed Talal

**Affiliations:** 1 Faculty of Computing and Meta-Technology (FKMT), Universiti Pendidikan Sultan Idris (UPSI), Tanjong Malim 35900, Perak, Malaysia; 2 Computer Techniques Engineering Department, Mazaya University College, Nassiriya 12733, Thi-Qar, Iraq; 3 SP Jain School of Global Management, Lidcombe, Sydney, NSW 2141, Australia; 4 Department of Business Administration, College of Administrative Science, The University of Mashreq, Baghdad 10021, Iraq; 5 Future Technology Research Center, National Yunlin University of Science and Technology, 123 University Road, Section 3, Douliou, Yunlin 64002, Taiwan; 6 Iraqi Commission for Computers and Informatics (ICCI), Baghdad 10022, Iraq; 7 Department of Computer Science, Kulliyyah of Information and Communication Technology, International Islamic University Malaysia, Kuala Lumpur 53100, Malaysia; 8 School of Business Administration, Faculty of Business Administration, Southwestern University of Finance and Economics, No. 555, Liutai Road, Wenjiang District, Chengdu 611130, China; 9 School of Mechanical, Medical, and Process Engineering, Queensland University of Technology, Brisbane, QLD 4000, Australia; 10 ARC Industrial Transformation Training Centre—Joint Biomechanics, Queensland University of Technology, Brisbane, QLD 4000, Australia; 11 Department of Electronic Engineering, Faculty of Electrical and Electronic Engineering, Universiti Tun Hussein Onn Malaysia (UTHM), Batu Pahat 86400, Malaysia

**Keywords:** emotion criteria, multi-chronic diseases, multi-criteria decision making, patients prioritisation, sensor criteria

## Abstract

An intelligent remote prioritization for patients with high-risk multiple chronic diseases is proposed in this research, based on emotion and sensory measurements and multi-criteria decision making. The methodology comprises two phases: (1) a case study is discussed through the adoption of a multi-criteria decision matrix for high-risk level patients; (2) the technique for reorganizing opinion order to interval levels (TROOIL) is modified by combining it with an extended fuzzy-weighted zero-inconsistency (FWZIC) method over fractional orthotriple fuzzy sets to address objective weighting issues associated with the original TROOIL. In the first hierarchy level, chronic heart disease is identified as the most important criterion, followed by emotion-based criteria in the second. The third hierarchy level shows that Peaks is identified as the most important sensor-based criterion and chest pain as the most important emotion criterion. Low blood pressure disease is identified as the most important criterion for patient prioritization, with the most severe cases being prioritized. The results are evaluated using systematic ranking and sensitivity analysis.

## 1. Introduction

There has been a surge of scientific works along with health and medical recommendations in recent years due to chronic diseases [1]. These diseases cause serious health concerns not only because of their severity but also due to the activity restrictions they impose on adults [2], the high healthcare cost they entail, and the long-term healthcare admissions [3]. According to statistics, more than 90% of adults have at least one chronic disease [4], and 65% to 85% have two or more [5]. The management of such diseases has become crucial, and the need for the continuous care provided by medical institutions has been increasing. In response to the latter, many technologies have been specifically integrated from all domains of science [6]; for example, computer science introduced the concept of telemedicine, enabling patient care from a distance [7]. This technological branch is defined as a remote medical practice where medical services are remotely provided, especially for patients in distant places and even during difficult times, such as the COVID-19 pandemic [8]. Telemedicine is also integrated with advanced computer technologies, such as the Internet of Things (IoT), not only to allow patients to remotely communicate with hospitals for consultations or non-emergency purposes but also to be managed and treated through connected body sensors [9]. They can aid in observing patients’ status and treating and monitoring their activities [10]. Some medical cases require immediate hospital admission and thus raise serious concerns for cases when the demand for healthcare services increases and causes unprecedented burdens on medical healthcare centres [11,12]. For such times, telemedicine-based triaging is introduced to sort the influx of patients to receive treatment according to their types, number of illnesses, and need and whether their treatment can be postponed or not. Patients with a chronic disease should definitely be prioritised, and those with more than one chronic disease at the same time should be prioritised over those with one. In response to these issues, two scientific interdisciplinary works have been introduced. Mohammed et al. [13] utilised decision science through multi-criteria decision-making (MCDM) to propose a novel patient-prioritisation methodology called the technique for reorganisation of opinion order to interval levels (TROOIL). The method considers patients with multiple chronic diseases (MCDs) in a real-time remote health monitoring system. Using a 500-patient dataset, the authors included three chronic diseases, namely (1) chronic heart disease (CHD), (2) high blood pressure, and (3) low blood pressure, and presented them in two groups of criteria measures. The first group was related to medical sensors, and the second group was related to textual emotions. On this basis, the authors proposed an approach with six steps: (1) transforming data into intervals, (2) generating a medical rule, (3) rule ordering, (4) expert rule validation, (5) data reorganization, and (6) criteria objective weighting and patient ranking. In the proposed method, patients with the most severe MCD were treated first on the basis of their highest priority levels, and the treatment of patients with less severe cases was delayed. In another extended work, Mohammed et al. [14] discussed the patient prioritisation problem for MCD with big data generated from multiple disease conditions, namely CHD and high and low blood pressure. The main contribution of their work is the utilisation of big data and various prioritisation approaches. Previous research works clearly show that the MCD patient prioritisation problem is considered in relation to only three types of diseases, and the methodologies based on decision science are used accordingly. However, in reality, these diseases, if not all, have their own characteristics and detailed criteria that can influence the prioritisation decision regarding patients. For instance, the sensor readings for CHD provide different indications and might affect the decision process. The same thing can be said for emotion-based criteria, which are also a part of the CHD. Decision science, particularly *MCDM*, has been utilised in previous studies [13,14] for patient prioritisation, and it requires various sets of criteria. These criteria are treated as if they are on the same hierarchy level. However, in reality, these criteria and their main groups and subgroups should be on different hierarchy levels, which is considered a case-study-related shortcoming. Thus, this research attempts to prioritise MCD patients in relation to their main criteria (sensor and emotion) while considering the difference in hierarchy level of the criteria using MCDM. Meanwhile, addressing this issue will not only consider the problem associated with the case study and the difference in hierarchy level but also the theoretical challenges associated with the MCDM approach, including the various levels of importance, the variation in the criteria, and their variety. The utilised MCDM method, i.e., TROOIL, which is an extended version of the hybrid DM and voting method (HDMVM) in previous studies, is theoretically enhanced to increase its robustness and make it suitable for addressing the prioritisation issue. In the MCDM context, the assignment of weights of the criteria is amongst the most important determinants in the prioritisation process, and in the context of MCDM, it can be performed either objectively or subjectively [15]. In the former, the raw data values are used to determine the importance of the criteria using methods such as entropy, while in the latter, experts’ opinions and knowledge are used to calculate the weights of the criteria [16]. Many methods have been developed towards that end, including the analytic hierarchy process [17] and the best worst method [18], which have proven their resilience amongst the subjective weighting approaches in the literature. Nevertheless, they cannot be considered ideal for weighting criteria of different hierarchy levels that are presented in this research. Therefore, a highly robust weighting methodology should be considered. Recently, the fuzzy-weighted zero-inconsistency (FWZIC) method was proposed [19]. This method assigns criteria weights with zero inconsistency over triangular fuzzy numbers [19], but owing to the complex nature of MCDM case studies and ambiguity and vagueness issues, FWZIC has been used under various fuzzy environments, including trapezoidal fuzzy numbers [20], Pythagorean fuzzy set, T-spherical fuzzy set (T-SFS) [19], and q-rung orthopair fuzzy sets [21]. All the aforementioned fuzzy environments have their fair share in addressing ambiguity and vagueness issues, but more work is needed to explore other non-used robust fuzzy sets. On this basis, the notion of similarity measurements for fractional orthotriple fuzzy sets (FOFS) and their applications were introduced in [22]. This method makes use of a more generalised form of SFS and picture fuzzy sets to cope with the awkward and complex information in fuzzy set (FS) theory. The FOFS is a more powerful technique with respect to the existing drawbacks because of its conditions (i.e., the sum of the *f* powers) of positive, neutral, and negative grades bounded to [0, 1]. In the FOFS, experts’ opinions do not have to be yes or no and can include some form of denial or abstinence. In many real-life situations, compared with other fuzzy sets, the FOFS is an essential instrument for accurately describing an object without complexity, uncertainty, or ambiguity. The FOFS has been used in various MCDM context cases, including the pattern recognition problem. Abosuliman et al. [23] established a three-way decision-making method on the basis of the FOF rough set model. Qiyas et al. [24] developed aggregation operators under the FOFS information to solve MCDM problems. Motivated by the advantages of FOFS, this work addressed the objective weighting issue by formulating a new subjective weighting method named fractional orthotriple fuzzy-weighted zero-inconsistency (FOFWZIC) that is combined with the TROOIL methodology to weigh criteria with different hierarchy levels, followed by MCD patient prioritisation.

## 2. Methodology

### 2.1. Identification

The proposed method is for MCD patient prioritisation. The new weighting method, FOFWZIC, is used with the MCD patient dataset adopted from the work of Mohammed et al. [13]. This dataset is considered for 500 MCD patients. Three diseases are reported in the dataset, i.e., CHD, high blood pressure disease (HBPD), and low-blood-pressure disease (LBPD) disease. CHD has two groups of sub-criteria, including the sensor sub-criteria (SpO2, BP, Peaks, QRS width, P-P, and ST El) and the emotion sub-criteria (Chest Pain, SH. Breath, Palip, and rest?). More details on the definition and meanings of these criteria are fully reported by Mohammed et al. [13]. These main criteria were measured according to the risk-level rules for each patient, meaning that a patient is assigned a risk level out of 5 (Risk, Urgent, Sick, Cold State, and Normal) for each of these diseases. Some consistent risk levels might be obvious, and some might vary across all the MCDs reported. Thus, *n* = 125 rules were generated, and patients were distributed using these rules for prioritisation. A total of *n* = 12/125 rules representing 12 different matrices were generated, and only the first rule was selected in this study for the proof-of-concept, that is, patients with risk level across all the MCDs for each disease. The (*n* = 38) patient matrix for rule 1 is presented in Table 1.

### 2.2. Development

#### 2.2.1. FOFWZIC-Based TROOIL

The patient prioritisation in this study is based on the FOFWZIC-based TROOIL. In this process, knowing the steps of the original TROOIL with which the FOFWZIC step was integrated is important to address the objective weight issue. The original TROOIL comprises six steps, and the last step (6th) explains how the FOFWZIC weighting approach is applied. The first 5 steps have been discussed in Mohammed et al. [13], and the details of the FOFWZIC integration are as follows. 

**Criteria weighting and alternatives ranking:** This step includes the weighting of criteria using the new FOFWZIC. According to the various rules created and the proof-of-concept matrix we adopted in this study, we integrated FOFWZIC to address the issue of objective criteria weighting. Then, the FOFWZIC-based TROOIL uses the new subjective criteria weighting in the prioritisation process. The steps involved in using FOFWZIC are explained in the following section. 

#### 2.2.2. Criteria Weighting by FOFWZIC

The new extended FOFWZIC is used in weighting the criteria used in this research. The following procedures are included in the process.

**Criteria definition:** The evaluation criteria are defined for MCD patients’ prioritisation through experts. These experts determine the importance levels for these criteria as presented in the following phase.

**Structured expert judgment (SEJ):** Different experts were selected based on their knowledge and expertise in CHDs. Upon the completion of the selection stage, the same experts were evaluated for data collection, and a five-point Likert scale was used in the process. Then, the linguistic scale terms were converted into their numerical equivalents (Table 2).

**Expert decision matrix (EDM).** The expert decision matrix is a crossover between the evaluation criteria and the SEJ panel. Every expert is intersected with each of the evaluation criteria to assign the level of importance.

**Application of a fuzzy membership function.** Upon the completion of the latter, the data are transformed into q-ROF-EDM for additional precision. For any fixed set *M*, the FOFS *p* on *M* is represented by the triple of mappings $\mu_{p}:M\to \left[0,1\right],v_{p}:M\to \left[0,1\right]\;\mathrm{and}\;s_{p}:M\to \left[0,1\right]$ as defined by Abosuliman et al. [23], where each $m\in M,\;\mu_{p}\left(m\right),\;v_{p}\left(m\right),\;\mathrm{and}\;s_{p}\left(m\right)$ represents the positive, neutral, and negative degrees, respectively, with the following conditions: $0\;\leq \mu_{p}{\left(m\right)}^{f}+v_{p}{\left(m\right)}^{f}+s_{p}{\left(m\right)}^{f}\leq 1,\;\left(f\geq 1\right)$. The FOFS *P* is expressed as Equation (1):

(1) $$P=\{\langle m,(\mu_{p}\left(m\right),v_{p}\left(m\right),s_{p}\left(m\right))\rangle |m\in M\}$$

The degree of hesitancy is presented in Equation (2).

(2) $$\pi_{p}\left(m\right)=\sqrt[{f}]{1-{\left(\mu_{p}\left(m\right)\right)}^{f}-{\left(v_{p}\left(m\right)\right)}^{f}-{\left(s_{p}\left(m\right)\right)}^{f}}$$

After applying the FOFNs (see Table 2) on the EDM and constructing the q-ROF-EDM, the fuzzy numbers are aggregated using the FOFS aggregation operator (Equation (3)) and the FOFS division operator (Equation (4)) [22].

(3) $$\mathrm{FOF}-\mathrm{AM}\left({\tilde{p}}_{1},{\tilde{p}}_{2},\ldots ,{\tilde{p}}_{n}\right)=\left\{{\left[1-\prod_{i=1}^{n}{\left(1-\mu_{{\tilde{p}}_{i}}^{f}\right)}^{\;}\right]}^{1/f}\;,\;\;\;\prod_{i=1}^{n}\nu_{{\tilde{p}}_{i}}^{\;}\;\;,\;\;\;\;{\left[\prod_{i=1}^{n}{\left(1-\mu_{{\tilde{p}}_{i}}^{f}\right)}^{\;}-\prod_{i=1}^{n}{\left(1-\mu_{{\tilde{p}}_{i}}^{f}-s_{{\tilde{p}}_{i}}^{f}\right)}^{\;}\right]}^{1/f}\right\}\;\;\;\;\;\;\;f\geq 1$$

(4) $$p_{1}⊘p_{2}=\left(\begin{array}{c} {\left(\frac{\left(\mu_{p1}^{f}(2-\mu_{p2}^{f}\right)}{1-\left(1-\mu_{p1}^{f}\right)\;\cdot \left(1-\mu_{p2}^{f}\right)}\right)}^{\frac{1}{f}}\;,\;\;\;\frac{{\left(\nu_{p1}^{f}-\nu_{p2}^{f}\right)}^{\frac{1}{f}}}{{\left(1-\nu_{p1}^{f}\cdot \;\nu_{p2}^{f}\right)}^{\frac{1}{f}}}\;\;,\\ \frac{{\left(s_{p1}^{f}-s_{p2}^{f}\right)}^{\frac{1}{f}}}{{\left(1-s_{p1}^{f}\cdot \;s_{p2}^{f}\right)}^{\frac{1}{f}}}\; \end{array}\right),$$

$$if\frac{\;\mu_{p2}^{f}}{\mu_{p1}^{f}}\;\geq \;\frac{1-s_{p2}^{f}}{1-s_{p1}^{f}\;}\;\frac{1+s_{p1}^{f}}{1+s_{p2}^{f}}\geq 1\;f\geq 1$$

Then, the mean values are calculated to obtain the final weights of the criterion. Each value of the q-ROF-EDM is computed by Equations (3) and (5).

(5) $${\tilde{P}}_{\;}\;⊘\lambda \begin{array}{c} =\left\{{\left(1-{\left(1-\mu_{{\tilde{P}}_{\;}}^{f}\right)}^{1/\lambda }\right)}^{1/f}\;,\;\;\nu_{{\tilde{P}}_{\;}}^{1/\lambda },\;\;s_{{\tilde{P}}_{\;}}^{1/\lambda }\;\right\} \end{array}\;for\;\lambda >0\;\mathrm{and}\;f\geq 1$$

Thereafter, the resulting fuzzy weights are defuzzied using Equation (6) to determine the final crisp weight values. For rescaling purposes, the weight of each criterion should be determined by the sum of all criterion weights.

(6) $$Score\;\left({\tilde{p}}_{\;}\right)=\mu_{{\tilde{p}}_{\;}}{}^{f}-\;\nu_{{\tilde{P}}_{\;}}^{f}-\;s_{{\tilde{p}}_{\;}}{}^{f}\;\;\;\;f\geq 1$$

After weighting the criteria using FOFWZIC, TROOIL utilises compromise ranking to prioritise the alternatives on the basis of the new weighted criteria. The details are presented below.

Compromised ranking is used to determine the ideal closest solution in which the patient with the highest risk level is prioritised. Alternatives are ranked as follows. (1) The alternatives’ compromise rank is determined using values (*S*, *R* and *Q*; *S_i_* and *R_i_*) from the weighted matrix. *S_i_* and *R*_i_ are derived using Equation (7).

(7) $$S_{i}=\sum_{j=1}^{n}\left(vm_{ij}\right),\;R_{i}=MAX_{j}\left(VM_{ij}\right),$$

where *S_i_* and *R_i_* are used to express the ranking measures. (2) The value of *Q_i_* is utilised in measuring the distance between the alternative and ideal solutions. The process is established using Equation (8).

(8) $$Q=\left[\frac{v\left(S_{I}-S^{*}\right)}{S^{-}+S^{*}}\right]+\left[\left(1-V\right)*\left(R_{I}-R^{*}\right)/\;R^{-}+R^{*}\right)],$$

where S* = min, *S_i_*, *S*^−^ = max *S_i_*, and R* = min *R_i_*, *R*^−^ = max *R_i_*.

The *v* value represents the weight strategy for the majority of the criteria. (3) The last step includes a group of alternatives which are sorted based on the *Q* score in ascending order. The lower the *Q* score is, the higher the priority of the alternative will be. Table 3 shows the parameters used and their description. 

## 3. Results and Discussion

Using FOFWZIC-based TROOIL, the results of criteria weighting for different hierarchy levels and patient prioritisations are presented. Referring to the methodological approach for FOFWZIC, the first step is identifying the set of criteria (i.e., sensor sub-criteria and the emotion sub-criteria) as discussed in Section 2.1. Then, as explained in the second step of FOFWZIC, the structured expert judgment was formulated based on data collected from the involved experts based on their knowledge and responses. This was accomplished using a five-point Likert scale questionnaire, which later was transformed into their numerical equivalent as discussed in Section 2.2.2. The next step was the membership role in FOFWZIC, using crisp values that are transformed into their fuzzy equivalents. Then, the fuzzification process is applied to measure the significance of the selected criteria. Three formulas are utilised in the process, i.e., (3)–(5). Subsequently, the calculation is performed for every criterion based on the experts′ mean preference. The final weight is determined using Equation (6). Thus, three expert preferences are utilised in the process without consistency. According to the philosophy of FOFS extension, various parameters are considered in the weighting process (i.e., *F* = 2, 3, 5, 7, 9). The different hierarchy weight levels are shown in Table 4.

According to the weight of the main criteria for the first hierarchy level of weights, CHD clearly has the highest importance across all the *F* parameters. LBPD follows in terms of weighting importance, and HBPD is the last. On the basis of various hierarchy levels, these criteria are transferred to the second layer to implement the weight changes within them as we use different hierarchy levels. The second-layer criteria of CHD are presented in Table 5. 

The table clearly shows that second hierarchy level weights are also changed for the importance of the CHD sub-criteria. The weighting results indicate that the emotion-based criteria outrank the sensor-based criteria in all the *F* parameters. Both sub-criteria (Sensor and Emotion) are also presented with their internal measures (i.e., sub-criteria for each) in the third-level hierarchy, and the results are shown in Table 6 and Table 7.

Within sensor-based criteria, sub-criterion Peaks has the highest importance weight across all *F* parameters, and SpO2 is the least important. These weights are presented for the second hierarchy CHD criteria in terms of sensors, and the second part, which relates to emotion, is presented in Table 7.

In the third layer and in terms of the emotion sub-criteria, Chest Pain is the most significant criterion among all the *F* parameters, followed by SH. Breath, and Palip is the least significant criterion. All the aforementioned criteria hierarchy levels present various importance levels, clearly showing how criteria change can vary across levels. However, these weights are scaled in the final criteria weighting results to be used alongside TROOIL in the prioritisation process. The details are presented in Table 8.

In the majority of the *F* parameters and across the different hierarchy levels, CHD, as a main criterion, received the highest weight. The combined weight is the result of both sensor- and emotion-based criteria across all the *F* parameters. The second most significant criterion is LBPD, and the least significant is HBPD. These weights are considered in FOFWZIC-based TROOIL along with the others in the prioritisation of MCD patients. Upon the completion of criteria weighting, MCD patients with a high risk level are prioritised using FOFWZIC-based TROOIL on the basis of the group decision-making results of the three experts involved in this research. Each expert used the fuzzy version utilised in the prioritisation process for all the *F* parameters of 2, 3, 5, 7, and 9, as shown in Table 9.

As shown in Table 9, the FOFWZIC-based TROOIL prioritisation results are discussed in the group decision-making (GDM) context according to the *F* parameters for 2, 3, 5, 7, and 9. The normal number represents the Q value, while the exponential number represents the final ranking of the patient. MCD patient 288 has the highest priority with a consistent Q score of (0.001) across all the *F* parameters. Patient (27) has the least priority and is ranked 38th with Q scores of 0.872, 0.879, 0.886, 0.888, and 0.889 across the *F* parameters. The table shows many consistent rankings with slightly different Q scores across all the *F* parameters. For instance, MCD patient *27* maintained the 38th ranking with a 44.73% score. MCD patients 31, 139, 175, 284, 315, and 426 maintained their rankings in *F* parameters 4 and 5, with a ranking consistency score of 15.78%. The same total number of MCD patients maintained their ranking in (3/5) *F* parameters with a ranking consistency score of 15.78%. MCD patients 141, 142, 143, 303, 316, 317, 319, and 423 only maintained their ranking in *F* parameters 4 and 5 with a 21.05% consistency score. The ranking varies across the *F* parameters because of the increase and decrease in the Q score. The 65.26% variance average was calculated for all the MCD patients’ prioritisation results across the *F* parameters. This variance clearly indicates how the Q score impacts the prioritisation of patients. This prioritisation is deemed the final one, and its evaluation is discussed in the following section.

## 4. Evaluation

The evaluation method in this research is based on systematic ranking and the sensitivity analysis. Both evaluation processes have been used in many MCDM context cases [25] to ensure the validity of the ranking based on the GDM context. The results of the systematic ranking on the basis of the scenarios are presented in Table 10.

The table clearly indicates that across all the *F* parameters, the mean value of a group is smaller than or equal to that of the following group. This observation confirms the validity of the ranking and the systematic ranking concept. In our case study and according to the results, our ranking is systematic. Another evaluation method that utilises sensitivity analysis is adopted in this research. Sensitivity analysis measures the weight changes and their effect on the ranking (e.g., *F* = 2) of a parameter over five scenarios [26] using Equation (9).

(9) $$w_{c}=\left(1-w_{s}\right)\times \left(w_{c}^{o}/W_{c}^{0}\right)=w_{c}^{o}-\Delta x\alpha_{c}$$

where

- $w_{s}$ is the highest significant contribution,
- $w_{c}^{o}$ represents the original weight values computed using the FOFWZIC method,
- $W_{c}^{0}$ is the sum of the original weights for the changing criteria weight values,
- Δx is the range of the changes applied to the weight values of the criteria, representing the limit values of the LLBT criterion, which are −0.353≤Δx≤0.647.

The elasticity coefficient ($\alpha_{c}$) was used to compute the relative offset of all the other criteria weights over the criterion with the highest significant contribution (i.e., the LBPD criterion with a value of 0.353 in the *F* = 2 parameter). The rationale behind considering LBPD as the most significant criterion despite having different hierarchy levels is that when the final weight of CHD was compared with the other main criteria, i.e., LPBD and HPBD, CHD’s weight was not maintained in CHD alone but distributed over its sub-criteria from the third layer for the emotion and sensor criteria as shown in Table 7. Therefore, that weight was used in the final prioritisation process. The elasticity coefficient ($\alpha_{c}$) results are presented in Table 11.

The interval of −0.353≤Δx≤0.647 was divided into five scenarios and produced new weight values, as shown in Table 12.

The weights generated by sensitivity analysis were used in assessing the MCD patient prioritisation in terms of risk level. Five scenarios were utilised in the process, with only patients 7 and 38 alternatives maintaining their FOFWZIC-based TROOIL with a score of 18.42% across all five scenarios. Patient 288 was given first priority, followed by patient *176*. The other patients to be prioritised were patients 315, 425, 15, 299, and 27, ranking 31st, 35th, 36th, 37th, and 38th, respectively. Next was the maintained ranking in four scenarios; in that regard, the majority, with 65.87% of alternatives, were presented, including patients 432, 320, 304, 144, 32, 16, 287, 284, 175, 430, 318, 319, 316, 283, 143, 140, 31, 28, 429, 426, 317, 427, 423, 141, and 139, who ranked 3rd–3rd, 17th–19th, 23rd–30th, and 32nd–34th, respectively. The last group of alternatives, including patients 142, 431, 428, 303, 300, and 424, were ranked 14th–16th and 20th–22nd, respectively. Finally, the correlations of the five scenarios were checked using Spearman’s rank correlation coefficient [21]. In general, the highest correlation was observed for S1 and S3 with a value of 1.00, followed by S2 with 0.9997, S4 with 0.9986 and S5 with 0.9032. 

## 5. Conclusions

The criteria for different hierarchy levels affect the case study in this research, and the best solutions for this issue, involve multi-layer weighting. Like in previous research that used the original TROOIL, the theoretical problem of objective weighting was encountered in this research. To address the theoretical weighting issue, a highly robust weighting methodology that produces a highly accurate weighting was integrated. Many methods from the literature were explored, but FWZIC is the most prominent in terms of replacing objective criteria weights with zero-inconsistency subjective weights, which is the most pressing issue in this research. In this study, FWZIC was extended to an uncertainty-free fuzzy environment. Thus, the notion of similarity measurements for FOFSs was integrated with FWZIC to handle the awkward and complex information in fuzzy set theory to accurately describe an object without complexity, uncertainty, or ambiguity. The methodology in the proposed work included two main phases. First, the MCD patient risk-level matrix was identified and selected as the proof-of-concept, followed by the development phase. In this phase, the sequential steps of the FWZIC-based TROOIL were applied, especially the replacement of the objective weight. In the evaluation stage, sensitivity analysis was performed to confirm the robustness of the proposed method. However, this study has two main limitations that can be addressed in future work. The first limitation is the reliance on the first rule, the risk-level MCD patient, as the proof-of-concept for the prioritisation problem. Other rules should be considered in future research. Another limitation is the use of only one Likert scale. Comparisons between these results and those produced when using other scales are warranted. In the future, we will consider extending FWZIC and TROOIL with different fuzzy types to address the vagueness issue. Finally, both methods can be applied as an integrated approach or having the two methods separately used, where FWZIC can be utilized for weighting the evaluation criteria and TROOIL for ranking and prioritizing the alternatives for multi-decision-making problems.

## Figures and Tables

**Table 1:** Decision Matrix.

P. No.	HBPD	LBPD	CHD									
			Sensor						Emotion			
			SpO2	BP	Peaks	QRS Width	P-P	ST El.	Chest Pain	SH. Breath	Palip	Rest?
15	4	4	0	4	0	0	0	0	1	1	1	0
16	4	4	0	4	0	0	0	0	1	1	1	1
27	4	4	1	4	0	0	0	0	1	0	1	0
28	4	4	1	4	0	0	0	0	1	0	1	1
31	4	4	1	4	0	0	0	0	1	1	1	0
32	4	4	1	4	0	0	0	0	1	1	1	1
139	4	4	2	4	0	0	0	0	1	0	1	0
140	4	4	2	4	0	0	0	0	1	0	1	1
141	4	4	2	4	0	0	0	0	1	1	0	0
142	4	4	2	4	0	0	0	0	1	1	0	1
143	4	4	2	4	0	0	0	0	1	1	1	0
144	4	4	2	4	0	0	0	0	1	1	1	1
175	4	4	1	4	1	1	0	1	1	1	1	0
176	4	4	1	4	1	1	0	1	1	1	1	1
283	4	4	2	4	1	1	0	1	1	0	1	0
284	4	4	2	4	1	1	0	1	1	0	1	1
287	4	4	2	4	1	1	0	1	1	1	1	0
288	4	4	2	4	1	1	0	1	1	1	1	1
299	4	4	0	4	0	0	1	0	1	0	1	0
300	4	4	0	4	0	0	1	0	1	0	1	1
303	4	4	0	4	0	0	1	0	1	1	1	0
304	4	4	0	4	0	0	1	0	1	1	1	1
315	4	4	1	4	0	0	1	0	1	0	1	0
316	4	4	1	4	0	0	1	0	1	0	1	1
317	4	4	1	4	0	0	1	0	1	1	0	0
318	4	4	1	4	0	0	1	0	1	1	0	1
319	4	4	1	4	0	0	1	0	1	1	1	0
320	4	4	1	4	0	0	1	0	1	1	1	1
423	4	4	2	4	0	0	1	0	0	1	1	0
424	4	4	2	4	0	0	1	0	0	1	1	1
425	4	4	2	4	0	0	1	0	1	0	0	0
426	4	4	2	4	0	0	1	0	1	0	0	1
427	4	4	2	4	0	0	1	0	1	0	1	0
428	4	4	2	4	0	0	1	0	1	0	1	1
429	4	4	2	4	0	0	1	0	1	1	0	0
430	4	4	2	4	0	0	1	0	1	1	0	1
431	4	4	2	4	0	0	1	0	1	1	1	0
432	4	4	2	4	0	0	1	0	1	1	1	1

The values represented in these criteria represent the following risk levels: 4 = Risk, 3 = Urgent, 2 = Sick, 1 = Cold State, and 0 = Normal. The nature of all the criteria is beneficial; that is, the higher the number assigned to a patient for each MCD is, the higher the priority for treatment given to the patient will be.

**Table 2:** Linguistic Terms, Numerical Scoring.

Numerical Scale	Linguistic Scale	FOFNs		
		μ	v	s
1	Not Important	0.15	0.85	0.1
2	Low Important	0.25	0.75	0.2
3	Medium Importance	0.55	0.5	0.25
4	Important	0.75	0.25	0.2
5	Very Important	0.85	0.15	0.1

**Table 3:** Parameters Used.

Symbol	Description
$\mu_{p}$	Positive degree
$v_{p}$	Negative degree
$s_{p}$	Neutral degree
f	Fractional power
$\pi_{p}$	Hesitancy degree
FOF−AM	Fractional orthopair fuzzy arithmetic mean aggregation
$S_{i}$	Ranking measure
$R_{i}$	Ranking measures

**Table 4:** First Layer Criteria Weighting.

*F* Parameter		*F* = 2	*F* = 3	*F* = 5	*F* = 7	*F* = 9
	Criteria					
HBPD		0.1731	0.2089	0.2374	0.2386	0.2378
LBPD		0.3534	0.3337	0.3011	0.2923	0.2901
CHD		0.4734	0.4573	0.4613	0.468	0.4720

**Table 5:** Second-Layer Criteria.

*F* Parameter		*F* = 2	*F* = 3	*F* = 5	*F* = 7	*F* = 9
	Criteria					
CHD	Sensor	0.3945	0.3978	0.3736	0.3558	0.3487
	Emotion	0.6054	0.6021	0.6263	0.6441	0.6512

**Table 6:** Third-Layer Criteria (Sensor).

*F* Parameters		*F* = 2	*F* = 3	*F* = 5	*F* = 7	*F* = 9
	Criteria					
Sensor Sub Criteria	SpO2	0.0643	0.1020	0.1445	0.1536	0.1558
	BP	0.2045	0.1968	0.1895	0.1892	0.1903
	Peaks	0.2147	0.2005	0.1866	0.1817	0.1792
	QRS width	0.2132	0.1929	0.1730	0.1658	0.1624
	P-P	0.1844	0.1721	0.1575	0.1557	0.1562
	ST El.	0.1187	0.1354	0.1486	0.1537	0.1558

**Table 7:** Third-Layer Criteria (Emotion).

Criteria		*F* = 2	*F* = 3	*F* = 5	*F* = 7	*F* = 9
	*F* Parameter					
Emotion Sub Criteria	Chest Pain	0.2753	0.2673	0.2682	0.2719	0.2751
	SH. Breath	0.2579	0.2555	0.2515	0.2503	0.2511
	Palip	0.2094	0.2269	0.2367	0.2379	0.236
	rest?	0.257	0.2501	0.2434	0.2397	0.2370

**Table 8:** Criteria Weighting Results.

Criteria			*F* = 2	*F* = 3	*F* = 5	*F* = 7	*F* = 9
		*F* Parameter					
HBPD			0.173	0.208	0.237	0.238	0.237
LBPD			0.353	0.333	0.301	0.292	0.290
CHD	Sensor	SpO2	0.0120	0.018	0.024	0.025	0.025
		BP	0.0382	0.035	0.032	0.031	0.031
		Peaks	0.0401	0.036	0.032	0.030	0.029
		QRS width	0.039	0.035	0.029	0.027	0.026
		P-P	0.0344	0.031	0.027	0.025	0.025
		ST El.	0.022	0.024	0.025	0.025	0.025
	Emotion	Chest Pain	0.078	0.073	0.077	0.082	0.084
		SH. Breath	0.073	0.070	0.072	0.075	0.077
		Palip	0.060	0.062	0.068	0.071	0.072
		Rest?	0.073	0.068	0.070	0.072	0.072

**Table 9:** Adopted Decision Matrix.

P. No.	*F* = 2 [Q] ^Rank^	*F* = 3 [Q] ^Rank^	*F* = 5 [Q] ^Rank^	*F* = 7 [Q] ^Rank^	*F* = 9 [Q] ^Rank^
15	[0.834] ^36^	[0.844] ^36^	[0.851] ^36^	[0.852] ^36^	[0.851] ^36^
16	[0.071] ^8^	[0.072] ^8^	[0.072] ^8^	[0.070] ^8^	[0.069] ^8^
27	[0.872] ^38^	[0.879] ^38^	[0.886] ^38^	[0.888] ^38^	[0.889] ^38^
28	[0.153] ^26^	[0.153] ^26^	[0.169] ^26^	[0.178] ^26^	[0.182] ^26^
31	[0.153] ^25^	[0.150] ^25^	[0.164] ^25^	[0.171] ^25^	[0.173] ^22^
32	[0.068] ^7^	[0.067] ^7^	[0.065] ^7^	[0.063] ^7^	[0.061] ^7^
139	[0.191] ^34^	[0.186] ^34^	[0.202] ^34^	[0.213] ^34^	[0.217] ^33^
140	[0.150] ^24^	[0.148] ^24^	[0.162] ^24^	[0.171] ^24^	[0.175] ^24^
141	[0.183] ^33^	[0.180] ^33^	[0.196] ^32^	[0.206] ^32^	[0.208] ^31^
142	[0.124] ^14^	[0.132] ^18^	[0.153] ^18^	[0.163] ^17^	[0.165] ^17^
143	[0.149] ^23^	[0.145] ^23^	[0.157] ^22^	[0.164] ^20^	[0.165] ^19^
144	[0.065] ^6^	[0.061] ^6^	[0.058] ^6^	[0.055] ^6^	[0.054] ^6^
175	[0.095] ^11^	[0.096] ^11^	[0.113] ^11^	[0.123] ^11^	[0.125] ^10^
176	[0.003] ^2^	[0.005] ^2^	[0.007] ^2^	[0.007] ^2^	[0.007] ^2^
283	[0.134] ^19^	[0.132] ^16^	[0.152] ^17^	[0.164] ^21^	[0.169] ^21^
284	[0.092] ^10^	[0.093] ^10^	[0.111] ^10^	[0.122] ^10^	[0.127] ^11^
287	[0.092] ^9^	[0.090] ^9^	[0.106] ^9^	[0.115] ^9^	[0.117] ^9^
288	[0.001] ^1^	[0.001] ^1^	[0.001] ^1^	[0.001] ^1^	[0.001] ^1^
299	[0.856] ^37^	[0.866] ^37^	[0.878] ^37^	[0.880] ^37^	[0.881] ^37^
300	[0.137] ^21^	[0.140] ^22^	[0.160] ^23^	[0.170] ^23^	[0.174] ^23^
303	[0.137] ^20^	[0.137] ^21^	[0.155] ^20^	[0.164] ^19^	[0.165] ^18^
304	[0.052] ^5^	[0.054] ^5^	[0.057] ^5^	[0.055] ^5^	[0.054] ^5^
315	[0.175] ^31^	[0.174] ^31^	[0.194] ^31^	[0.205] ^31^	[0.210] ^32^
316	[0.134] ^18^	[0.135] ^19^	[0.153] ^19^	[0.163] ^18^	[0.167] ^20^
317	[0.167] ^29^	[0.167] ^29^	[0.188] ^30^	[0.198] ^30^	[0.200] ^28^
318	[0.108] ^13^	[0.120] ^13^	[0.144] ^14^	[0.155] ^14^	[0.157] ^14^
319	[0.134] ^17^	[0.132] ^17^	[0.148] ^16^	[0.156] ^16^	[0.158] ^15^
320	[0.049] ^4^	[0.049] ^4^	[0.050] ^4^	[0.048] ^4^	[0.046] ^4^
423	[0.181] ^23^	[0.175] ^32^	[0.197] ^33^	[0.212] ^33^	[0.218] ^34^
424	[0.140] ^22^	[0.136] ^20^	[0.156] ^21^	[0.170] ^22^	[0.175] ^25^
425	[0.206] ^35^	[0.204] ^35^	[0.226] ^35^	[0.240] ^35^	[0.245] ^35^
426	[0.164] ^28^	[0.165] ^28^	[0.185] ^28^	[0.197] ^28^	[0.202] ^29^
427	[0.172] ^30^	[0.169] ^30^	[0.186] ^29^	[0.198] ^29^	[0.202] ^30^
428	[0.131] ^16^	[0.130] ^15^	[0.146] ^15^	[0.156] ^15^	[0.159] ^16^
429	[0.164] ^27^	[0.162] ^27^	[0.180] ^27^	[0.191] ^27^	[0.193] ^27^
430	[0.105] ^12^	[0.115] ^12^	[0.137] ^12^	[0.147] ^12^	[0.150] ^12^
431	[0.130] ^15^	[0.127] ^14^	[0.141] ^13^	[0.149] ^13^	[0.150] ^13^
432	[0.045] ^3^	[0.044] ^3^	[0.042] ^3^	[0.040] ^3^	[0.039] ^3^

**Table 10:** Systematic Ranking Results.

Group #	*F* = 2	*F* = 3	*F* = 5	*F* = 7	*F* = 9
	Mean Value				
Group 1	0.736111	0.736111	0.736111	0.736111	0.736111
Group 2	0.675926	0.689815	0.689815	0.675926	0.671296
Group 3	0.6	0.5875	0.5875	0.6	0.6
Group 4	0.520833	0.520833	0.520833	0.520833	0.525

**Table 11:** Elasticity Coefficient ($a_{c}$) For Changing Weights.

Criteria	HBPD	LBPD	CHD									
			Sensor						Emotion			
			SpO2	BP	Peaks	QRS Width	P-P	ST El.	Chest Pain	SH. Breath	Palip	Rest
*F* = 2 (*α_c_*)	0.26772	0.54657	0.01859	0.05909	0.06205	0.06162	0.05329	0.03431	0.12209	0.11435	0.09287	0.11403

**Table 12:** Sensitivity Analysis.

P. No.	FOFWZIC-Based TROOIL	S1	S2	S3	S4	S5
15	36	36	36	36	36	36
16	8	8	8	8	8	19
27	38	38	38	38	38	38
28	26	26	26	26	26	29
31	25	25	25	25	25	30
32	7	7	7	7	7	15
139	34	34	34	34	34	33
140	24	24	24	24	24	20
141	33	33	32	33	33	34
142	14	14	14	14	16	21
143	23	23	23	23	23	22
144	6	6	6	6	6	9
175	11	11	11	11	11	5
176	2	2	2	2	2	2
283	19	19	19	19	19	6
284	10	10	10	10	10	3
287	9	9	9	9	9	4
288	1	1	1	1	1	1
299	37	37	37	37	37	37
300	21	21	21	21	22	23
303	20	20	20	20	21	24
304	5	5	5	5	5	10
315	31	31	31	31	31	31
316	18	18	18	18	18	16
317	29	29	29	29	29	32
318	13	13	13	13	13	17
319	17	17	17	17	17	18
320	4	4	4	4	4	8
423	32	32	33	32	32	25
424	22	22	22	22	20	11
425	35	35	35	35	35	35
426	28	28	28	28	28	26
427	30	30	30	30	30	27
428	16	16	16	16	15	12
429	27	27	27	27	27	28
430	12	12	12	12	12	13
431	15	15	15	15	14	14
432	3	3	3	3	3	7

## Data Availability

Not applicable.
